# Effectiveness of an mHealth Intervention to Help Parents Prevent Early-Onset Alcohol Involvement: Findings from a Pilot of a Randomized Waitlist Control Trial

**DOI:** 10.1007/s11121-025-01875-y

**Published:** 2026-01-20

**Authors:** Nisha Gottfredson O’Shea, Marina Stranieri Pearsall, Melissa B. Gilkey, H. Luz McNaughton Reyes, Susan T. Ennett

**Affiliations:** 1 RTI International, 3040 E. Cornwallis Dr., Durham, NC 27709 USA; 2 Gillings School of Global Public Health, University of North Carolina at Chapel Hill, Chapel Hill, USA

**Keywords:** Early-onset alcohol use, Parenting interventions, Mobile health

## Abstract

A quarter of 11-year-old children in the USA have tried alcohol, typically provided by parents. Parents are the primary source of alcohol socialization for preteens, yet many are unaware of their influence and lack tools to discourage early-onset alcohol involvement (EOAI). We piloted *BIPAS Alcohol,* an mHealth intervention grounded in social cognitive theory, to prevent alcohol socialization. In this two-arm randomized waitlist control trial, 132 parents were randomized to receive *BIPAS Alcohol* immediately or after a 3-month delay. Self-reported outcomes were assessed at baseline and at 3 months. We analyzed intent-to-treat (ITT) effects using generalized linear models. We also tested moderation of intervention exposure by parental alcohol use frequency. *BIPAS Alcohol* improved parents’ alcohol prevention beliefs, attitudes, and behaviors, including parenting self-efficacy, permissive beliefs, communication with other caregivers, alcohol socialization, and allowing sips. In moderation analyses, parent alcohol use frequency was associated with weaker effects on alcohol access and stronger effects on allowance of sipping. Moderation analyses suggested stronger effects on permissive beliefs and allowance of sips and weaker effects on alcohol access among parents who drank more frequently. *BIPAS Alcohol* is a promising preventive intervention for delaying EOAI. Its long-term effectiveness should be confirmed using a larger, more diverse sample.

**Supplementary Information:**

The online version contains supplementary material available at 10.1007/s11121-025-01875-y.

Early-onset alcohol involvement (EOAI), defined as drinking before age 14, is a serious and prevalent risk behavior. Although rates of adolescent alcohol use have been declining since 2020, in 2024, nearly 20% of eighth-graders in the USA reported having had more than a sip of alcohol in their lifetime (Miech et al., 2025). EOAI is a robust predictor of alcohol problems during late adolescence: youth who begin drinking early are more likely to escalate rapidly to binge drinking and alcohol use disorder (AUD) than those who delay initiation (Aiken et al., 2020; Colder et al., 2018; Murphy et al., 2021; Staff & Maggs, 2020; Yap et al., 2017). Data from the U.S. National Longitudinal Alcohol Epidemiological Survey suggest that 40% of children with EOAI were diagnosed with AUD later in life, whereas only 10% of those who delayed drinking until age 21 or later had an AUD diagnosis (Grant & Dawson, 1998).

Beyond its contribution to risk for AUD later in life, alcohol is the leading cause of death for those under age 21 (Hingson & Zha, 2009). Adolescent alcohol involvement disrupts brain development, impairing cognitive performance and emotion regulation, and it increases the risk of mental health issues such as depression, social isolation, and suicidal ideation (Hussong et al., 2011; Schilling et al., 2009; Skogen et al., 2016; Windle et al., 1992). Furthermore, EOAI sets children on a cyclical process of selection into, and influence from, risk-taking peer groups (Ennett et al., 2006, 2008; Hussong, 2002). In turn, affiliation with risk-taking peers is related to many concurrent negative health outcomes, including binge drinking (DeWit et al., 2000; Ellickson et al., 2001; Zucker et al., 2008), risky sexual behavior (Hingson & Kenkel, 2004; Windle et al., 1992), aggressive and violent behavior (Hingson & Kenkel, 2004; Reyes et al., 2015; Windle et al., 1992), smoking and other drug use (Ennett et al., 2006), and academic problems (Hingson & Kenkel, 2004).

Parents are children’s primary source of alcohol-related social modeling and information during childhood and the preteen years, shaping normative beliefs and attitudes that influence EOAI (Latendresse et al., 2008; van der Vorst et al., 2005; Wills & Cleary, 1999). Children whose parents consume alcohol are more likely to engage in EOAI; however, this risk is fully mitigated when parents clearly communicate disapproval of child alcohol use—even of small sips from family members’ alcoholic beverages (Ennett et al., 2013). Indeed, EOAI usually begins in a family context, often with small amounts of alcohol, or sips, provided to children by their parents (Komro et al.,  2007; van der Kruk et al., 2023); Wadolowski et al., 2015; Warner & White, 2003). Estimates suggest that approximately 33%–35% of U.S. parents allow children to sip alcohol (Colder et al., 2018); Ennett et al., 2016; Jackson et al., 2012; Jackson, Colby, et al., 2015). Many parents who allow their children to sip alcohol endorse statements such as, “If they drink small amounts of alcohol at home, children can learn to be responsible drinkers,” “Letting children find out what alcohol tastes like will make them less likely to want to taste it again,” and “If parents don’t let children try alcohol at least once, children will be more tempted by alcohol as a’forbidden fruit’” (Jackson et al., 2012). These pro-sipping beliefs are misguided. Multiple well-controlled, prospective studies have identified no protective effects and several negative effects of parents’ providing sips of alcohol to their children (Aiken et al., 2020; Clare et al., 2020; Colder et al., 2018; Hawkins et al., 1997; Jackson et al., 2015; Murphy et al., 2021).

Parents are central to child alcohol socialization—not only through modeling use, but also by communicating alcohol-related expectations, countering media messages, discussing risks, setting alcohol-specific family rules, monitoring children’s behavior at home and with peers, and, in some cases, allowing or providing sips of alcohol to their children (Ryan et al., 2010; Stephenson et al., 2023; Yap et al., 2017). Given their centrality, parents should be the primary targets for EOAI preventive interventions.

A randomized controlled trial of *Mysteries, Max, and Me* (*MMM*), a family-based intervention designed to reduce parental modeling of alcohol use and other permissive alcohol socialization behaviors, demonstrated significant and sustained reductions in child susceptibility to alcohol use 4 years after program exposure. Notably, *MMM* had the strongest beneficial effects among parents who initially held more permissive beliefs—those presumed to be the least receptive to program messaging (Jackson et al., 2016). *MMM* was delivered to parents via monthly activity booklets mailed to parents of third-graders for 6 months, with booster booklets sent at 12 and 24 months post-baseline. Although the *MMM* program was found to be highly effective, its reach is limited due to its outdated and resource-intensive delivery method.

## The Current Study

**Fig. 1:**
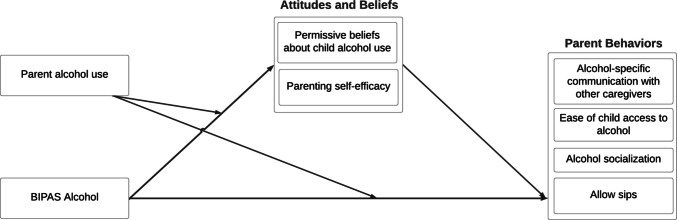
Conceptual model of intervention mechanisms of action Alt text: Figure showing mediation of the effects of BIPAS Alcohol exposure on parent behaviors via parental attitudes and beliefs. Parent alcohol use moderates the paths between intervention exposure and attitudes and beliefs. It also moderates the direct path from intervention exposure to parent behavior outcomes

This manuscript presents estimates of the efficacy of our updated version of *MMM*, which we call *Better-Informed Parents Keeping Adolescents Safe from Alcohol (BIPAS Alcohol*), based on intent-to-treat (ITT) analyses from a pilot study conducted with *N* = 132 parents of rising sixth-graders. The primary focus is on proximal intervention targets—namely, parental attitudes, beliefs, and behaviors (see Fig. 1 for the conceptual model). We assessed ITT effects on our pre-specified primary outcome: permissive parental beliefs, as well as other proximal outcomes, including allowance of sips, perceived ease of alcohol access, alcohol socialization behaviors, parenting self-efficacy, and communication with other caregivers. Because parental alcohol use was used as a tailoring variable that affected the content of messages that parents received, we conducted an exploratory analysis to examine whether intervention effects were moderated by parental alcohol use.

## Method

### Participants

Participants were parents of rising middle school children residing in central North Carolina. Participants were eligible if they met the following inclusion criteria: (1) were parents or guardians (henceforth, “parents”) of a 10- to 12-year-old child with whom they lived at least part time, (2) used a smartphone or other device with text messaging capabilities, (3) were able to complete study activities in English, and (4) were willing to be randomized to receive the intervention immediately or after a waiting period. Parents who had more than one age-eligible child were asked to select the youngest child to be the primary focus of their study activities.

We recruited parents via electronic school newsletters for parents delivered to all public elementary schools in Durham, Chatham, and Orange counties, as well as the Charlotte-Mecklenburg (city–county) school district (54% of recruited parents), participant referrals (23%), emails disseminated through university electronic mailing lists (17%), social media posts (5%), and flyers posted at local pediatric clinics (1%). Study advertisements instructed interested individuals to scan a QR code that directed them to an online pre-screening questionnaire or to contact the study team via email. Research staff reviewed questionnaires and scheduled telephone calls with parents for further screening. During each phone call, research staff provided study details, confirmed eligibility (including confirming the presence of a child of the appropriate age by speaking with the child), and described next steps for enrollment. Parents who wished to enroll completed an electronic consent and child permission form. The study protocol was approved by the University of North Carolina Institutional Review Board (study #22–0101) and is available on ClinicalTrials.gov (ID #NCT05520333).

Recruitment occurred from May through November 2023, until we reached the sample size necessary to have 80% power to detect a medium effect (Cohen’s *d* =.5) under a two-sided hypothesis test with α =.05, assuming 5% loss of information due to attrition.

### Procedure

Participants were randomized with equal probability to receive the intervention immediately (*n* = 69) or after 3 months (*n* = 63). A binomial random number generator was used to create a list of 0 s and 1 s indicating trial arm before the trial started; the study manager used treatment arm assignment to program the text message schedule. All participants received the 3-month intervention during June 2023–July 2024. Parents in both arms completed digital surveys on the Qualtrics platform at the time of enrollment, 3 months after enrollment (*N* = 66 [96%] in the intervention-first arm and *N* = 59 [94%] in the waitlist arm).

Surveys were sent to parents via text message, followed by two text or email reminders for nonresponders. Eighty-eight percent of completed surveys were answered on the day that they were delivered; all completed parent and child surveys were finished in under 7 days, except that one 3-month parent survey for a parent in the intervention-first group was completed after 11 days. Participants received a $25 gift card after completion of each survey.

### Intervention Description: BIPAS Alcohol

**Fig. 2:**
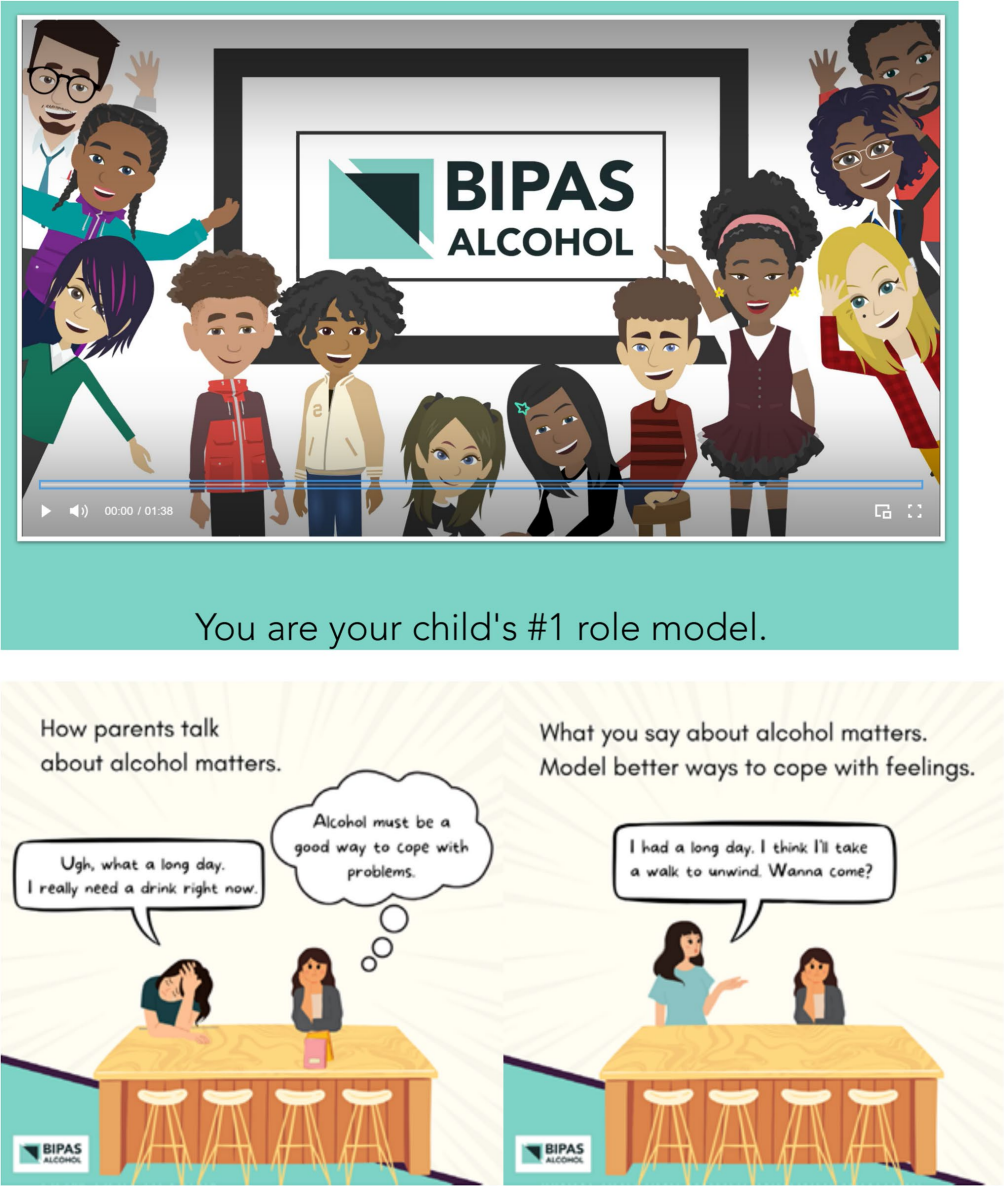
Screenshot of video on bipasalcohol.com (top) and series of two graphic text messages delivered to parents (bottom) Alt text: Top—A screenshot showing a diverse group of pre-adolescents and parents in cartoon format. The characters are smiling and waving. They are surrounding the BIPAS Alcohol logo. The bottom of the screen says “You are your child’s #1 role model.” Bottom—A cartoon with two panels. The first panel says: “How parents talk about alcohol matters.” There is a picture of a mother and her daughter. The mother says: “Ugh, what a long day. I really need a drink right now.” The daughter is thinking: “Alcohol must be a good way for me to cope with problems.” The next panel says: “What you say about alcohol matters. Model better ways to cope with feelings.” The same mother and daughter are pictured. The mother is saying: “I had a long day. I think I’ll take a walk to unwind. Wanna come?”

To address limitations in the delivery format and timing of the original intervention, our team adapted *MMM* into a digital format targeting slightly older children. The resulting intervention, *BIPAS Alcohol*, was developed in collaboration with a team that specializes in graphic design for health communication. *MMM* content was converted into 50 brief text messages with linked website content designed to be inclusive, engaging, informative, and digitally appealing (see Fig. 2, www.bipasalcohol.com).

Grounded in social cognitive theory (Bandura, 2001) and principles of persuasion (Cialdini, 1993), *BIPAS Alcohol* proceeds along the following progression of themes and topics: (1) parents as role models (building parental self-efficacy and awareness of parental influence; moral engagement); (2) presentation of evidence of harms caused by EOAI and parent-supplied alcohol (risk awareness); (3) skills and strategies for alcohol-specific communication with children, including setting clear, alcohol-specific rules; (4) the role of peers (monitoring, communication with children and other parents and caregivers); (5) information on alcohol in the media and strategies for countering positive alcohol messaging; and (6) a template for developing a family agreement about alcohol.

With a Flesch-Kincaid grade level of 6.3 and a reading ease score of 71.8, messages were designed to be accessible to parents with low literacy. Most messages included a graphic or linked to a video animation illustrating key themes on the *BIPAS Alcohol* website. We developed text messages and website content iteratively with qualitative input from 11 parents of rising middle schoolers. Participants provided feedback on tone, visual appeal, clarity, and persuasiveness. On the basis of this input, comic-style visuals were created, and a user-friendly website was developed to house supplemental materials and animations.

Text messages were tailored based on parent alcohol use at screening. For example, nondrinking parents received messages such as, “Even if you don’t have alcohol in your home, talk to other adults in your child’s life and make sure they understand why having sips of alcohol is not okay.” Parents who reported regular alcohol use were coached in how to communicate differences between adult and child alcohol use: “What’s the best way to respond when kids ask why you or other adults drink alcohol? Here is an idea: ‘Drinking affects kids more than adults, and I want to help keep you safe and healthy’”.

### Measures

#### Outcome Variables

Dependent variables were assessed at baseline and 3 months post-baseline. For outcomes assessed using multi-item scales, we used confirmatory factor analysis (CFA) with multivariate weighted least squares estimator to assess model fit and to output factor score estimates for use in our primary analyses of intervention effects. CFAs accounted for nesting of repeated observations within reporter by using robust standard errors. We report the CFA-based omega reliability for each latent construct (Flora, 2020).

#### Permissive Beliefs (Ennett et al., 2013; Primary Outcome)

Parents were asked to rate their level of agreement from *strongly disagree* (0) to *strongly agree* (3) with each of the following statements: “If they drink small amounts of alcohol at home, children can learn how to be responsible drinkers,” “Letting children find out what alcohol tastes like will make them less likely to want to taste it again,” “Letting preteens have sips or tastes of alcohol at home is a safe way to introduce them to alcohol,” “If parents tell children they are not allowed to have any alcohol, they will only want it more,” and “Preteens who sip small amounts of alcohol at home with parents will be less likely to experiment with risky drinking in middle school.” To avoid problems with estimation, we collapsed *strongly agree* and *agree* responses for “bad taste,” “safe way,” and “less risky” because of low endorsement rates in these categories. Model fit was exceptional: χ^2^(5) = 5.15, *p* =.40; confirmatory fit index (CFI) = 1.00; Tucker-Lewis index (TLI) = 1.00; root mean squared error of approximation (RMSEA) =.01; standardized root mean squared residual (SRMR) =.01. Standardized factor loadings ranged from.67 to.96. Omega reliability for this scale was.86.

#### Allow Sips of Alcohol

Parents answered *yes* (1) or *no* (0) to the question, “Is your child ever allowed to sip alcohol in your home?”.

#### Parental Alcohol Socialization Behaviors

Parents were asked, “How often do you serve or order your child “mocktails” or drinks that look like adult drinks, but that don’t have alcohol in them?”, “How often does your child see you with an alcoholic drink?”, “How often do you ask your child to bring you a drink?”, and “How often do you let your child know that you disapprove of the way alcohol is being used by a character in a TV show or movie?” Response options ranged from *never* (0) to *very often* (3). Responses *very often* and *somewhat often* were collapsed for the “mocktails” item due to low endorsement rates; all options other than *never* were also collapsed for the “child bring drink” item due to sparseness. Standardized factor loadings ranged from.47 to.77. Omega reliability for this scale was .60.

#### Parenting Self-Efficacy (Ennett et al., 2013)

Parents were asked the following questions: “How strongly do you agree or disagree that you can influence whether your child has any alcohol between now and when they turn 13 years old?” Response options ranged from strongly disagree (0) to *strongly agree* (3), later recoded as *strongly agree* (1) or *anything else* (0). “How confident are you that setting rules for your child about alcohol use would keep them from drinking when they are in middle school?” and “How confident are you about talking to your child about alcohol use?” Response options ranged from *not confident* (0) to *very confident* (3), later recoded as *not confident* (0) or *confident* (1) due to item sparseness. “If one of your child’s friends was sipping or drinking alcohol, do you think your child would let you know about it?” Response options ranged from *definitely not* (0) to *definitely yes* (3). The *definitely not* and *probably not* response options were later collapsed due to item sparseness. Model fit was acceptable: χ^2^(2) = 4.28, *p* =.12; CFI =.97; TLI =.92; RMSEA =.06; SRMR =.06. Standardized factor loadings ranged from .67 to .96. Omega reliability for this scale was .61.

#### Ease of Access (Schulenberg et al., 2017)

Parents were asked, “How easy or hard would it be right now for your child to get even a small amount of beer, wine, or other alcohol at home?” Response options ranged from *very easy* (0) to *very hard* (3).

#### Alcohol Communication With Other Caregivers

Parents were asked to report whether, in the past 30 days, they had “Talked to another parent about underaged alcohol use,” “Talked to another adult in your child’s life about limiting access to alcohol,” or “Talked to parents of your child’s friends about whether your child will be around alcohol while they are at their friend’s house.” Response options were no (0) or yes (1). Standardized factor loadings ranged from.69 to.86. Omega reliability for this scale was .59.

#### Demographics and Moderator

In the baseline survey, parents self-reported their age, relationship to the child, level of education, and racial identity, as well as the age and gender of their child. Parents also reported their own alcohol use, which was used to test moderation of intervention effects. Alcohol use frequency was assessed with the item, “How often did you have a drink containing alcohol in the past 30 days.” Response options were *never* (0), *monthly or less* (1), *2–4 times per month* (2), 2–3 times per week (3), and *4 or more times per week* (4).

#### Data Analysis

##### Preliminary Multiple Imputation, Data Management, and Confirmatory Factor Models

Our analytic models relied on full information maximum likelihood estimation, which results in unbiased parameter estimates under the assumption of random missingness (conditional on covariates included in the model; Graham, 2009). However, we used multivariate weighted least squares estimation to generate factor score estimates prior to estimating predictive models. We also had access to additional auxiliary variables not used in the analytic models that might aid in informing missing data values, so we used the R package jomo to conduct multilevel multiple imputation with joint modeling for all missing data (Collins et al., 2001; Gottfredson et al., 2017; Quartagno & Carpenter, 2023). We generated 20 imputed datasets that were used for all subsequent analyses. Next, we inspected and reported response distributions and polychoric correlations for all measures overall and by treatment arm. Sparse response categories were collapsed to facilitate estimation. Mplus software was used to estimate CFAs and output factor score estimates, which were exported to R.

##### ITT Effects of BIPAS Alcohol on Parental Attitudes, Beliefs, and Behaviors

We used generalized linear models to compare change from baseline (*t* = 0) to 3 months post-baseline (*t* = 3) for parents who received the intervention during that period and for parents who were still on the waitlist during that period. We used a variation of Eq. 1 for all outcomes except allowance of sips because the latter was binary:

1 $$\left({y}_{3i}-{y}_{0i}\right) = {b}_{0}+{b}_{1}{y}_{0i}+{b}_{2}T{x}_{i}+{r}_{ti}$$

We modeled allowance of sips using the model shown in Eq. 2:

2 $$ln\left({~}^{p(allo{w}_{3i})}\!\left/ \!{~}_{1-p(allo{w}_{3i})}\right.\right) = {b}_{0}+{b}_{1}{y}_{0i}+{b}_{2}T{x}_{i}$$

All models controlled for baseline values to avoid bias due to regression to the mean.

##### Effect Modification by Frequency of Parental Alcohol Use

We tested whether the effects of exposure to *BIPAS Alcohol* on intervention outcomes varied as a function of frequency of parental alcohol use by including parental alcohol use frequency as a main effect and including it in an interaction term with treatment assignment. We probed significant interactions to obtain simple slopes conditional on frequency of parental drinking for the effects of *BIPAS Alcohol* exposure (Preacher et al., 2006).

We applied the Benjamini–Hochberg correction to maintain a 5% Type I error rate for the main effects and for the interaction effects involving *BIPAS Alcohol* assignment.

## Results

Sample descriptive statistics are reported in Table 1. There were no significant differences in participant characteristics by intervention arm except for parent education: parents in the waitlist control group were significantly less likely than those in the intervention arm to have more than a high school education. Nearly all parents reported drinking at least occasionally; most reported consuming one or two drinks per drinking occasion. Participants tended to be mothers, to be White, and to have a graduate or professional degree.

**Table 1:** Participant characteristics

Characteristic	Total (*N* = 132) *n* (%) or M (SD)	Intervention First (*N* = 69) *n* (%) or M (SD)	Waitlist Control (*N* = 63) *n* (%) or M (SD)
Child age	11.06 (.77)	10.95 (.81)	11.20 (.69)
Child gender			
Boy	70 (53%)	38 (55%)	32 (51%)
Girl	56 (42%)	29 (42%)	27 (43%)
Other/prefer not to say	6 (5%)	2 (3%)	4 (6%)
Parent relationship to child			
Mother	115 (87%)	61 (88%)	54 (86%)
Father	16 (12%)	8 (12%)	8 (13%)
Other guardian	1 (1%)	0 (0%)	1 (2%)
Parent age	43.02 (6.75)	42.37 (6.90)	43.77 (6.56)
Parent race/ethnicity			
White	116 (89%)	63 (90%)	53 (87%)
Black/African American	4 (3%)	3 (4%)	1 (2%)
Asian/Pacific Islander	8 (6%)	3 (4%)	5 (8%)
Hispanic	6 (5%)	3 (4%)	3 (5%)
Other	3 (2%)	1 (1%)	2 (3%)
Parent educational attainment			
High school or less	7 (5%)	1 (1%)	6 (10%)
Some college	4 (3%)	1 (1%)	3 (5%)
Bachelor’s degree	31 (24%)	18 (26%)	13 (21%)
Graduate or professional degree	89 (68%)	50 (71%)	39 (64%)
Parent alcohol frequency			
Never	3 (3%)	1 (2%)	2 (4%)
Monthly or less	24 (23%)	14 (25%)	10 (21%)
2–4 times per month	29 (28%)	19 (34%)	10 (21%)
2–3 times per week	31 (30%)	14 (25%)	17 (36%)
4 or more times per month	16 (16%)	8 (14%)	8 (17%)
Parent alcohol quantity per drinking occasion (drinkers only)			
1–2 drinks	104 (93%)	53 (88%)	51 (98%)
3–4 drinks	6 (5%)	5 (8%)	1 (2%)
5 + drinks	2 (2%)	2 (4%)	0 (0%)

Descriptive statistics are based on raw (non-imputed) baseline data

Outcome variable descriptive statistics and change scores based on raw, non-imputed data are reported in Table 2.

**Table 2:** Parent outcomes by intervention arm and study phase

Outcome variable	Baseline (0 months)		3 months post-baseline			
	Intervention first *n* = 69	Waitlist control *n* = 63	Intervention first *n* = 66		Waitlist control *n* = 59	
	Baseline	Baseline	Post-intervention	Δ from 0 months^a^	Second baseline	Δ from 0 months^a^
Allow sips: *N* (%)	15 (22%)	12 (19%)	10 (15%)	− 5 (− 7%)	17 (29%)	5 (10%)
Permissive beliefs: M (SD)	.35 (.91)	.27 (.81)	−.23 (.82)	−.62 (.75)	.26 (.76)	−.03 (.50)
Parenting self-efficacy: M (SD)	−.10 (.60)	−.10 (.59)	−.03 (.76)	.05 (.61)	−.11 (.66)	.02 (.68)
Ease (difficulty) of access: M (SD)	1.14 (1.10)	.97 (1.09)	1.34 (1.11)	.20 (.64)	1.03 (1.14)	.05 (.71)
Caregiver communication: M (SD)	−.17 (.41)	−.08 (.50)	.31 (.70)	.48 (.73)	−.10 (.49)	−.05 (.55)
Alcohol socialization: M (SD)	.14 (.70)	.05 (.79)	.01 (.71)	−.16 (.48)	.04 (.82)	.00 (.47)

Permissive beliefs, parenting self-efficacy, and caregiver communication are factor score estimates. Descriptive statistics reported in this table are from raw, non-imputed data

^a^Change from month 0 to 3 months post-baseline was compared in analyses

### Model Results

Table 3 contains ITT model results. Unstandardized regression coefficients are provided in the table. To provide information about effect sizes, we report standardized coefficients (β) or odds ratios in the text.

**Table 3:** Generalized linear models

	Main effects model			Interaction model		
	Estimate	95% LL	95% UL	Estimate	95% LL	95% UL
Δ parenting self-efficacy						
Intercept	− 11.93	− 12.92	− 10.94	− 11.99	− 13.07	− 10.90
Baseline self-efficacy	−.07	−.36	.23	−.06	−.36	.24
Tx group	12.13	11.84	12.43	12.15	11.57	12.74
Parent alcohol frequency				.02	−.15	.19
Parent alcohol frequency*Tx group				−.01	−.26	.24
Δ permissive beliefs						
Intercept	.08	.04	F.11	−.10	−.16	−.04
Baseline permissive beliefs	−.35	−.38	−.33	−.34	−.36	−.31
Tx group	−.55	−.59	−.50	−.18	−.26	−.09
Parent alcohol frequency				.08	.06	.11
Parent alcohol frequency*Tx group				−.18	−.22	−.15
Δ ease (difficulty) of access						
Intercept	.23	.19	.27	.59	.51	.67
Baseline ease of access	−.18	−.20	−.15	−.26	−.29	−.24
Tx group	.17	.12	.22	.28	.19	.38
Parent alcohol frequency				−.14	−.16	−.11
Parent alcohol frequency*Tx group				−.05	−.09	−.01
Δ communication with other caregivers						
Intercept	−.09	−.12	−.05	−.04	−.10	.02
Baseline communication	−.61	−.65	−.56	−.60	−.65	−.55
Tx group	.48	.43	.52	.43	.35	.52
Parent alcohol frequency				−.02	−.05	.00
Parent alcohol frequency*Tx group				.02	−.02	.06
Δ alcohol socialization						
Intercept	.01	−.01	.04	−.42	−.47	−.37
Baseline socialization	−.19	−.21	−.16	−.44	−.47	−.41
Tx group	−.15	−.18	−.12	−.09	−.15	−.03
Parent alcohol frequency				.22	.19	.24
Parent alcohol frequency*Tx group				−.01	−.04	.01
Allow sips^a^						
Intercept	1.19	1.17	1.22	1.09	1.06	1.13
Baseline allowance of sips	1.78	1.72	1.83	1.75	1.70	1.81
Tx group	.86	.84	.88	.92	.87	.96
Parent alcohol frequency				1.04	1.03	1.06
Parent alcohol frequency*Tx group				.97	.95	.99

*LL* lower limit, *Tx* treatment, *UL* upper limit

^a^Estimates and confidence bounds have been exponentiated for “Allow sips” outcomes to obtain odds ratios

#### Permissive Beliefs

When we controlled for baseline permissive beliefs, parents exposed to the intervention had a significantly greater reduction in permissive beliefs at the 3-month follow-up (β =  −.72). This effect was moderated by parental alcohol use frequency. Controlling for baseline permissive beliefs, the difference between the mean change in permissive beliefs between parents in the treatment and control groups was β =  −.25 among parents who reported no drinking and β =  − 1.27 for parents who reported weekly drinking.

#### Allow Sips

When we controlled for whether they allowed sips at baseline, parents who received the intervention were less likely than parents in the control condition to allow sips at the 3-month follow-up. This effect was moderated by parent alcohol use frequency. Among parents who reported no alcohol use, intervention assignment was associated with OR =.92 (95% CI.88,.96); among parents who drank at least weekly, intervention assignment was associated with OR =.82 (95% CI.77,.85).

#### Parenting Self-Efficacy

When we controlled for baseline levels of parenting self-efficacy, parents who received the *BIPAS Alcohol* intervention had substantially greater improvements in parenting self-efficacy during the first 3 months of the study than parents who were on the waitlist to receive the intervention (β = 1.97). Parent alcohol use frequency was not a statistically significant moderator of this effect.

#### Ease of Access

When we controlled for baseline ease of access, parents who received the *BIPAS Alcohol* intervention reported that it became significantly more difficult for their children to obtain alcohol (β =.25). This effect was moderated by parental alcohol use frequency such that controlling for baseline ease of access, there was a β =.42 unit difference in change between the intervention and control arms for parents who reported no alcohol use; this effect was not significant among parents who reported weekly drinking (β =.12).

#### Communication with Other Caregivers

When we controlled for baseline levels of communication, parents in the intervention arm reported a significantly greater change in communication with other caregivers than parents in the control group. Parental drinking status was not a statistically significant moderator of this effect.

#### Alcohol Socialization

When we controlled for baseline alcohol socialization, parents who were exposed to the intervention reported a significantly greater decrease in alcohol socialization behaviors at the 3-month follow-up (β =.68). Parental drinking status was not a statistically significant moderator of this effect.

## Discussion

Analyses supported our hypotheses that parental exposure to the universal, low-intensity, digital *BIPAS Alcohol* intervention reduces permissive beliefs, alcohol socialization behaviors, child ease of alcohol access, and allowance of child sipping, and it increases parenting self-efficacy and alcohol-specific communication with both children and other caregivers. Moderation analyses show that, although the intervention is effective for all or most of the target outcomes regardless of parental drinking frequency, there are some differences in the mechanisms of action across levels of parental alcohol use: parents who drink alcohol more frequently develop less permissive attitudes and behaviors as a result of intervention exposure.

*BIPAS Alcohol* is innovative in substance and form. The program directly targets parents as key influencers of their children’s alcohol use, recognizing that some parents may be promoting EOAI, even if inadvertently, through permissive practices. The program’s substantive focus on permissive beliefs and behaviors, particularly allowance of child alcohol use (including sips), is a novel basis for targeting parents as change agents. Because it includes content related to avoiding alcohol socialization outside the home, focusing more on this content for parents who report not drinking, the program is universally applicable. Also novel is our focus on parents of children in the preparatory stage of alcohol use development, as they are making the transition to middle school—alcohol prevention programs have overwhelmingly focused on older adolescents in the experimental stage of alcohol use development. With rates of ever drinking doubling as children transition from elementary to middle school, the middle school transition is a key intervention point, and early interventions to prevent EOAI must target the family (Donovan, 2007; Miech et al., 2025).

Parents are children’s primary source of social modeling and information about alcohol use prior to the teen years (Latendresse et al., 2008; van der Vorst et al., 2005; Wills & Cleary, 1999). Thus, the family environment is an important driver of EOAI that can and should be leveraged for prevention. Face-to-face interventions such as *Coping Power* (Lochman & Wells, 2002), *Guiding Good Choices* (Mason et al., 2003), and S*trengthening Families* (Kumpfer & Magalhães, 2018) are widely recognized as efficacious for preventing adolescent substance use among the families who participate (Elliott et al., 2020). These in-person programs, however, face dissemination and implementation barriers that limit reach (Leslie et al., 2016). Some of the main barriers that prevent parent engagement in these types of interventions include difficulty obtaining transportation and childcare, busy schedules, and perceived stigma (Hansen et al., 2019). As there have been increasing calls for wide-reaching, universal public health solutions to improve youth outcomes with family-based interventions (Metzler et al., 2012; Vimpani, 2005), digital delivery of interventions has emerged as an approach that reduces barriers to entry and the cost of service provision while increasing reach and scalability (Calam et al., 2008). Digital interventions like *BIPAS Alcohol* can be delivered at any time and are a low-cost way to widely reach parents via a communication method that they are already using.

### Study Limitations and Recommendations for Future Research

The generalizability of study findings is limited by the lack of diversity in the sample: the majority of participants were White mothers with a high education level and higher-than-average levels of alcohol use (e.g., 52% of U.S. adults report drinking at least once a month, whereas 74% of parents in our sample reported drinking at least once a month [National Institute on Alcohol Abuse and Alcoholism, 2025]). Participants may have been motivated by a desire to engage in a study about preventing adolescent alcohol use and therefore may have responded more favorably to the intervention. Although our higher socioeconomic sample limited generalizability, it is also the case that White parents with higher socioeconomic status tend to drink more than other parents, and parent drinking is a strong risk factor for EOAI (Donovan & Molina, 2011; Obot et al., 2001). Thus, in this case, the intervention reached a high-risk sample and was effective for this group. It is plausible that prevention in this high-risk group could have cascading prevention effects by reducing alcohol access for their children’s peers or through parental communication channels.

This study was limited by the short length of follow-up and its reliance on parent self-report. We focused on parent-reported outcomes because the follow-up window was not sufficient to detect meaningful differences in child alcohol use among rising middle school students, particularly given the modest sample size (*N* = 132). Because of this, it is difficult to discern the practical significance of the intervention effects in terms of impacts on child alcohol use behavior. Longer-term studies are needed to assess EOAI and test whether parent exposure to *BIPAS Alcohol* leads to delayed or reduced alcohol initiation. These studies should include child reports on parent behaviors to validate parent self-report.

Despite these limitations, we are encouraged by the sustained effects of *MMM*, the intervention on which *BIPAS Alcohol* is based, which demonstrated reductions in child susceptibility to alcohol use up to 4 years post-intervention (Jackson et al., 2016).

## Conclusion

*BIPAS Alcohol* is a low-cost, universal digital intervention that reduces barriers to equitable, universal implementation of family-based preventive interventions by delivering intervention content directly to parents’ mobile devices in digestible chunks, incrementally over time. Because it eliminates barriers to implementation, *BIPAS Alcohol* has the potential to serve families across social and environmental contexts. *BIPAS Alcohol* reaches parents at a critical point for preventing EOAI, when children are entering middle school. The results presented in this manuscript show that *BIPAS Alcohol* is efficacious in a selected sample of parents. The next step is to conduct a larger-scale study with a large, diverse sample of parents to assess heterogeneity in program effects and to identify implementation barriers that need to be overcome prior to scaling up the intervention.

## Supplementary Information

Below is the link to the electronic supplementary material.

ESM 1 (32.8 KB DOCX)

## Data Availability

See NIAAA for data archive.

## Declarations

### Clinical Trials

ClinicalTrials.gov (ID #NCT05520333), August 25, 2022.
